# The Application of Automatic Identification System Information and PSO-LSTM Neural Network in CRI Prediction

**DOI:** 10.1155/2022/8699322

**Published:** 2022-03-24

**Authors:** Wei Zhou, Yun Li, Yingjie Xiao, Jian Zheng

**Affiliations:** ^1^Merchant Marine College, Shanghai Maritime University, Shanghai 201306, China; ^2^Shanghai High-level Local University Innovation Team (Maritime Safety & Technical Support), Shanghai 201306, China; ^3^College of Transport and Communications, Shanghai Maritime University, Shanghai 201306, China

## Abstract

Considering that collision accidents happen sometimes, it is necessary to predict the collision risk to ensure navigation safety. With the information construction in maritime and the popularity of automatic identification system application, it is more convenient to obtain ship navigation dynamics. How to obtain ship encounter dynamic parameters through automatic identification system information, assess ship collision risk, find out dangerous target ships, and give early warning and guarantee for ship navigation safety, is a problem that scholars have been studying. As an index to measure the degree of ship collision risk, CRI, namely, collision risk index, is usually obtained by calculating ship encounter parameters and comprehensive analysis. There are many factors that affect CRI, and the values of many parameters depend on expert judgment. The corresponding CRI has nonlinear and complex characteristics, which is highly correlated with the time sequence. In order to enhance the prediction accuracy and efficiency, PSO-LSTM neural network is applied in the paper to predict CRI. Experiments show that PSO-LSTM neural network can effectively predict collision risk and provide a reference for navigation safety.

## 1. Introduction

Ship collision accidents are the main type of marine accident, and are more likely to occur in port areas with dense ship traffic. It is usually accompanied by casualties, property losses and marine environmental pollution, which often attracts extensive attention. For example, according to Reference [1], at approximately pm 7:50, Jan.6th, 2018, the Panamanian tanker “Sanchi” collided with the bulk carrier “CF CRYSTAL,” resulting in ship sinking, casualties and oil pollution. Due to the serious consequences, people pay special attention to ship collision, which is assessed, forecast and forewarning, so as to avoid the occurrence of collision accidents and reduce the related losses. Some scholars have proposed the ship collision risk index (CRI), which is widely used to quantify the degree of collision risk [2]. Generally, parameters such as DCPA and TCPA are used to quantify ship collision risk [3–5]. Then, the ship domain, which is a safe area around the own ship, is applied to measure the collision risk [6–9]. In Reference [10], once the target ship enters the own ship's domain, there may be collision risk. With the popularity of AIS data and the convenience of data acquisition, more and more new methods have been used to evaluate ship collision risk. In References [11, 12], AIS data are used to measure the risk level of ship collision in the waters. Reference [13] uses evidence theory to evaluate ship collision analysis. According to Reference [14], the artificial neural network method is used to quantify ship collision analysis. Fuzzy inference method is used to calculate ship collision risk by Reference [15]. In Reference [16], a quantitative assessment algorithm based on SVM technology can obtain collision risk according to ship sailing status. From Reference [17], images are constructed by AIS data, and then a method based on CNN and image recognition is used to classify ship collision risks. In short, with the trend of intelligent navigation, more and more intelligent technologies have begun to be applied in the field of marine transportation to serve and ensure the safety of ship navigation. Considering that the ship driver not only needs to know the current collision risk of the ship, but also wants to predict the possible risk, it is necessary to predict the collision risk. Taking an example as follows: reference [18] deduces an ordered probabilistic regression model to study perceived collision risk. In Reference [19], the domain method is adopted to carry out dynamic risk warning for ship encounter process.

LSTM neural network, as an excellent deep learning model, has been widely used. An automatic collision avoidance algorithm based on LSTM for ship continuous motion space is proposed in reference [20], and the experiment shows that it is effective. PSO can optimize hyperparameters of LSTM. Therefore, PSO-LSTM neural network can be used for more accurate prediction. For instance, according to reference [21], ship traffic flow prediction is currently faced with problems of high randomness, many influencing factors and low accuracy, so PSO-LSTM prediction model is proposed to improve the prediction accuracy. In reference [22], due to the high randomness and complexity of ships sailing at sea, it is difficult to make accurate prediction. PSO-LSTM is applied in forecast ship's comprehensive posture. Considering that the measurement of collision risk depends on expert judgment, the ship collision risk index has the characteristics of nonlinear, random, empirical, fuzzy and complex, etc. Meanwhile, as ship encounter process parameters are a time sequence with correlation, this paper proposes using PSO-LSTM model for prediction analysis.

The main contribution of this paper is as follows: PSO-LSTM model is applied to predict collision risk. The feasibility of the prediction method is analyzed through the design of single-ship and multi-ship encounter situation experiments. The change characteristics of CRI during ship encounter are studied and visualized. AIS data is collected by ship maneuverability simulator test to ensure the correctness, integrity and synchronization of data, so as to ensure the accuracy of CRI prediction data sources. By comparing with pilot's experience in BRM practical operation test, the conformity of CRI and its prediction with the actual situation is verified.

The rest of this paper is organized as follows: in Section 2, the quantitative methods of collision risk are discussed, including ship collision parameters and fuzzy membership quantification methods. In Section 3, the basic principles of PSO algorithm and LSTM model are introduced, and the ship collision risk prediction model using PSO-LSTM is established. Simulation experiments are taken in Section 4. Finally, the conclusion is drawn in Section 5.

## 2. Quantitative Method of Ship Collision Risk

At present, ship collision risk quantification methods include the collision theory analytical calculation method, ship domain method, fuzzy mathematics method, artificial neural network method, data statistics method, evidence theory method, support vector machine method, etc. Because collision risk index is a fuzzy concept, the fuzzy mathematical method is widely used. In this paper, AIS data is used to calculate ship encounter parameters. On this basis, CRI is obtained by the fuzzy membership degree, so as to obtain the time sequence data set required for ship collision risk prediction.

### 2.1. Calculation of Ship Encounter Parameters

**Figure 1:**
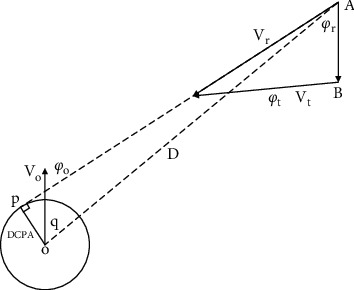
Geometric relation diagram of ship encounter parameters.

In the process of ship encounter, DCAP, TCPA and other parameters are usually used for quantitative analysis, so as to quantify collision risk. Suppose that the own ship's geographic coordinate is (*x*_*o*_, *y*_*o*_), the speed is *V*_*O*_, and the course is *φ*_*o*_; the geographical coordinate of the target ship is (*x*_*t*_, *y*_*t*_), the speed is *V*_*t*_, and the course is *φ*_*t*_. The geometric relation of the parameters is shown in Figure 1, and the calculation formula of relevant parameters [23] is as follows: where *D* is the relative distance (unit: nautical mile), *V*_*r*_ is the relative ship speed (unit: knot), *φ*_*r*_ is the relative heading (unit: degree), and *q* is the relative bearing (unit: degree). The value of the arc-tangent function needs to be based on the position relationship, and the range is [0,360°]. In Reference [24], DCPA is the minimum encounter distance (unit: nautical mile), and TCPA is the minimum encounter time (unit: minute). Considering the calculation requirements of the membership function, DCPA and TCPA are greater than zero in this paper, and absolute values are taken when there are negative values.


(1) $$\begin{array}{c} D=\sqrt{{\left(x_{t}-x_{o}\right)}^{2}+{\left(y_{t}-y_{o}\right)}^{2}},\\ V_{r}=V_{O}\sqrt{1+{\left(\frac{V_{t}}{V_{O}}\right)}^{2}-\frac{2V_{t}}{V_{O}}\cos\left(\varphi_{t}-\varphi_{o}\right)},\\ \phi_{r}=\cos^{-1}\left(\frac{V_{O}-V_{t}\cos\left(\varphi_{t}-\varphi_{o}\right)}{V_{r}}\right),\\ q=\arctan\left(\frac{x_{t}-x_{o}}{y_{t}-y_{o}}\right),\\ \text{DCPA}=D.\;\sin\left(\phi_{r}-q-\pi \right),\\ \text{TCPA}=D.\;\cos\frac{\left(\phi_{r}-q-\pi \right)}{V_{r}}, \end{array}$$

DCPA and TCPA are the main parameters used to measure collision risk. When DCPA and TCPA are less than the safety value, there may be a collision. However, to obtain the collision risk, it is not enough to consider only DCPA and TCPA. Other factors, such as distance and relative orientation, etc., should also be considered.

### 2.2. CRI and Membership Function

CRI is used to measure ship collision risk. Usually, the value range can be [0, 1]. The higher the value, the more dangerous it is. DCPA, TCPA, *D*, *q* and *k* (ratio of the speeds of the other and own ship) can be used as the constituent index to measure collision risk, which is obtained by weighting the membership degree in fuzzy mathematics. This paper refers to the membership function in Reference [13] and the weighting coefficient which is the survey results of the experts in Reference [25]. The membership function and weighting coefficient of the above parameters are as follows:

(2) $$\begin{array}{c} \mu \left(\text{DCPA}\right)=\left\{\begin{array}{cc} 1,\; & \text{DCPA}\leq d_{1},\\ \frac{1}{2}-\frac{1}{2}\text{sin}\left[\frac{\pi }{d_{2}-d_{1}}\left(\text{DCPA}-\frac{d_{1}+d_{2}}{2}\right)\right], & d_{1}<\text{DCPA}<d_{2},\\ 0, & d_{2}\leq \text{DCPA}, \end{array}\right) \end{array}$$

(3) $$\begin{array}{c} \mu \left(\text{TCPA}\right)=\left\{\begin{array}{cc} 1, & \text{TCPA}\leq t_{1},\\ {\left(\frac{t_{2}-\text{TCPA}}{t_{2}-t_{1}}\right)}^{2}, & \;t_{1}<\text{TCPA}\leq t_{2},\\ 0, & \;t_{2}<\text{TCPA}, \end{array}\right) \end{array}$$

(4) $$\begin{array}{c} \mu \left(D\right)=\left\{\begin{array}{cc} 1, & 0<D\leq D_{1},\\ {\left(\frac{D_{2}-D}{D_{2}-D_{1}}\right)}^{2}, & \;D_{1}<D\leq D_{2},\\ 0, & D_{2}<D, \end{array}\right) \end{array}$$

(5) $$\begin{array}{c} \mu \left(q\right)=\frac{17}{44}\left[\cos\left(q-19^{{}^{\circ}}\right)+\sqrt{\cos^{2}\left(q-19^{{}^{\circ}}\right)+\frac{440}{289}}\;\right], \end{array}$$

(6) $$\begin{array}{c} \mu \left(k\right)=\frac{1}{1+{\left(k_{0}/k\right)}^{2}}, \end{array}$$

where *d*_1_ is the minimal safe encounter distance, and *d*_2_ is the absolute safe encounter distance in (2). *t*_1_ and *t*_2_ are the time to reach the corresponding positions of *D*_1_ and *D*_2_  by relative motion in (3). *D*_1_ is the latest steering distance, and *D*_2_  is the moving boundary in (4). The above parameters are calculated according to Reference [12] and Reference [23]. In (5), when *q*  is equal to  19°; the collision danger degree is the highest. *k*_0_ is usually taken as one in (6).

Weighting the membership degree of the above parameters, the collision risk index formula is as follows:

(7) $$\begin{array}{c} \text{CRI}=\mu \left(\text{DCPA}\right)*w_{1}+\mu \left(\text{TCPA}\right)*w_{2}+\mu \left(D\right)*w_{3}+\mu \left(q\right)*w_{4}+\mu \left(K\right)*w_{5}, \end{array}$$

where the values of weight coefficients *w*_1_, *w*_2_, *w*_3_, *w*_4_, and *w*_5_ are 0.36, 0.32, 0.14, 0.10, and 0.08, respectively.

## 3. Collision Risk Prediction Model

### 3.1. LSTM Model

**Figure 2:**
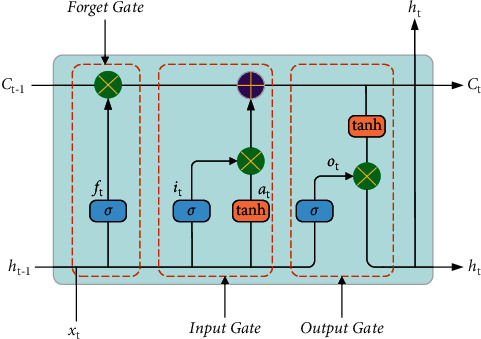
Basic structural unit of LSTM.

LSTM neural network, namely long and short memory neural network, is an improved network of RNN [26]. According to Reference [27], LSTM model can remember past information and store it for a long time by adding additional memory units, which has strong generalization ability, good learning ability for both large and small data sets, and strong advantages in dealing with nonlinear problems. LSTM model can reflect the time dependence and correlation in time sequence data of AIS date and predict ship collision risk. The main structure of LSTM model has three gates. Its basic structure is shown in Figure 2.

The forget gate *f*_*t*_ determines the information to be discarded and retained according to the unit state *C*_*t*−1_ at the previous moment, and the input *x*_*t*_ determines the value to be updated through *σ* and tanh respectively and generates new candidate values for updating [27]. The value after the updating operation will be updated together with the Forget Gate *f*_*t*_, and the updated unit state *C*_*t*_ is computed with the tanh function and Output Gate *o*_*t*_ and then outputs *h*_*t*_ [28]. The state update equation of LSTM basic unit are equations (8)–(13).

(8) $$\begin{array}{c} f_{t}=\sigma \left(W_{f}.\left[h_{t-1},x_{t}\right]\right)+b_{f}, \end{array}$$

(9) $$\begin{array}{c} i_{t}=\sigma \left(W_{f}.\left[h_{t-1},x_{t}\right]\right)+b_{i}, \end{array}$$

(10) $$\begin{array}{c} a_{t}=\tanh\left(W_{c}.\left[h_{t-1},x_{t}\right]\right)+b_{c}, \end{array}$$

(11) $$\begin{array}{c} C_{t}=f_{t}.C_{t-1}+i_{t}.a_{t}, \end{array}$$

(12) $$\begin{array}{c} o_{\text{t}}=\sigma \left(W_{o}.\left[h_{\text{t}-1},x_{\text{t}}\right]\right)+b_{o}, \end{array}$$

(13) $$\begin{array}{c} h_{t}=o_{t}.\;\tanh\left(C_{t}\right), \end{array}$$

where *x*_*t*_ is the input at time t; *f*_*t*_, *i*_*t*_, *o*_*t*_ represent forget, input and output gate respectively. *a*_*t*_ is the input node state at the corresponding moment; *C*_*t*−1_ and *C*_*t*_ are the unit states at the corresponding moment. *h*_*t*−1_ and *h*_*t*_ are the outputs at corresponding moment. *σ* is the sigmoid activation function, and tanh is a hyperbolic tangent function [28]; *W*_*f*_, *W*_*i*_, *W*_*c*_, and *W*_*o*_ and *b*_*f*_, *b*_*i*_, *b*_*c*_, and *b*_*o*_ are the corresponding weight matrices and offsetting vectors.

### 3.2. PSO Algorithm

PSO algorithm is an optimization technology proposed according to the foraging behavior of birds, which updates the formula with speed to make the particles in the population constantly close to the historical optimal value [29]. Firstly, the particle is given an initial velocity and position information in the solution space in a random way, and then the local and global optimal solutions are tracked and their velocities and positions are updated in time through the defined Fitness function. In this process, the Fitness value is calculated through each iteration, so as to achieve the set target Fitness value, and then the optimal solution can be obtained.

In the search space, several particles form a population, and after the t iteration, the velocity and position of particles are formed, which are represented by *X*_*i*,*t*_ and *V*_*i*,*t*_ respectively. During the search process, the position and velocity are updated constantly, and two optimal solutions are formed: one is the individual extremum *pbest*_*i*_, and the other is the global optimal solution *gbest*_*i*_. In the process of searching for the optimal solution, the particle updating formulas of velocity and position [30] are (14) and (15), respectively.

(14) $$\begin{array}{c} X_{i,t+1}=\omega X_{i,t}+c_{1}\text{rand}\left(pbest-X_{i,t}\right)+c_{2}\text{rand}\left(gbest-X_{i,t}\right), \end{array}$$

(15) $$\begin{array}{c} X_{i,t+1}=X_{i,t}+\lambda V_{i,t+1}, \end{array}$$

where *ω* is the inertia factor; *c*_1_ and *c*_2_ are individual and global learning factors, respectively. *rand* is a random number in [0,1]. *λ* is the velocity coefficient.

### 3.3. PSO-LSTM Model for Collision Risk Prediction

**Figure 3:**
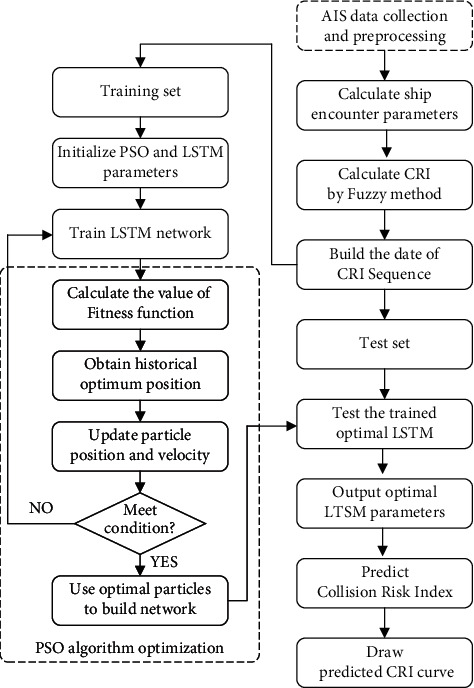
Flow chart of collision risk prediction by PSO-LSTM model.

As hyperparameters greatly impact on the results, PSO algorithm can improve the accuracy of LSTM model by optimizing parameters [31]. The flow chart of PSO-LSTM prediction model is shown in Figure 3. The steps are as follows:

**Step 1 .**

According to the AIS data, the ship motion parameters are obtained and the ship encounter parameters are calculated.

**Step 2 .**

CRI is obtained by calculating the membership function value and weight coefficient by fuzzy mathematics, and the training and test data are constructed according to the time sequence. Since the range of CRI is already [0, 1], normalization is not required.

**Step 3 .**

The particle swarm parameters are initialized, including the population size, maximum number of iterations, learning factor, particle positions and velocity ranges [32]. Meanwhile, the hyperparameters of LSTM neural network are initialized, including the number of hidden layer neurons, the learning rate, the maximum number of iterations of LSTM network, and the number of steps in the input layer.

**Step 4 .**

The LSTM model is established, which is trained with the data of the training set. The results are compared with the training set, and RMSE is used as the Fitness function in PSO algorithm. The calculation formula is as follows:

(16) $$\begin{array}{c} \text{RMSE}=\sqrt{\frac{1}{n}\overset{n}{\underset{i=1}{\sum }}{\left(\hat{Y_{i}}-Y_{i}\right)}^{2}}, \end{array}$$

where *Y*_*i* _ and $\hat{Y_{i}}$ are the real value of the CRI time sequence and the corresponding predicted value of the model respectively.

**Step 5 .**

The global optimal position *gbest* and local optimal position *pbest* are determined by the initial Fitness value of the particles [33], and they are regarded as the historical optimal positions. According to the speed and position of the updated particles, the corresponding particle Fitness value is calculated and then updated to improve the prediction accuracy.

**Step 6 .**

The termination condition is judged to be satisfied (the Fitness value of particles tends to be stable or reaches the maximum number of iterations with iteration [33]). If the termination condition is satisfied, optimal parameters are assigned to PSO-LSTM model; otherwise, step (4) is returned.

**Step 7 .**

PSO-LSTM prediction model is constructed from the optimal parameter values, which is applied to predict CRI of the test sets. The predicted result curves are drawn, and the ship collision risk is visualized.

## 4. Experiment

**Figure 4:**
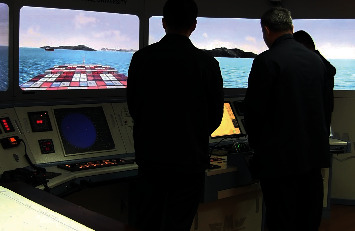
Pilot's actual operation in ship handling simulator.

This paper is based on AIS data of pilot's BRM (bridge resource management, which is included in the Mandatory Enforcement Part A of the STCW convention by IMO in 2010) practice test on ship handling simulator, and the actual operation site is shown in Figure 4. The main reasons for choosing the ship maneuvering simulator for the test are as follows: first, different encounter situations are simulated by setting the same environmental parameters. In the case of the same other conditions, interference is eliminated so as to compare the difference of different encounter situations. Second, the AIS data collected by the simulator have a complete structure and high data frequency, which can well solve the problem of a lack of AIS data and time synchronization. All these are conducive to better process analysis. Finally, experienced pilots perform practical operation experiments, which can not only ensure the professionalism of the experiment, but also ask pilots how they feel in the process of ship encounter, which can be verified and compared with the results of the test data analysis. In addition, the disadvantages of the simulator test also need to be explained, mainly as follows: the environmental conditions in a certain water area are relatively fixed, and are not as complex as the actual water area. The pilot's sense of urgency for dangerous situations is not as strong as the actual situation, which has a certain influence on the judgment of collision risk.

Through data acquisition and processing, ship encounter parameters and fuzzy collision risk index are calculated, PSO-LSTM network is trained to predict CRI, and single-ship encounter situation and multi-ship encounter situation tests are carried out. Considering that disadvantage of PSO-LSTM neural network is that it requires a long period of training, it is necessary to set reasonable parameters to avoid PSO optimization parameter setting too large search range and iteration times, to improve operation efficiency. Therefore, during the experiments, the PSO parameters are set as follows: the learning factors *c*_1_ and *c*_2_ are both set to 2, the range of the inertia factor *ω* is from 0.8 to 1.2, the maximum number of evolution is 20, and the size of the particle population is 5.

The selected water area for the test is the approach channel of Shanghai Yang Shan Port. The main reasons for the test in this area are as follows: Shanghai Yang Shan Port is a world-famous container terminal, and its inbound and outbound channels have certain typicality. At the same time, due to the large scale of navigable ships, the navigation safety of their waters is also a concern. The parameters of the own ship and target ships are shown in Table 1. The environmental parameters are shown in Table 2, which need to be marked, because environmental conditions, especially strong winds and waves, will affect the navigation difficulty of ships, affect the pilots' judgment of collision risk, and then affect the generation and prediction results of training datasets.

### 4.1. Single-Ship Encounter Experiment

**Figure 5:**
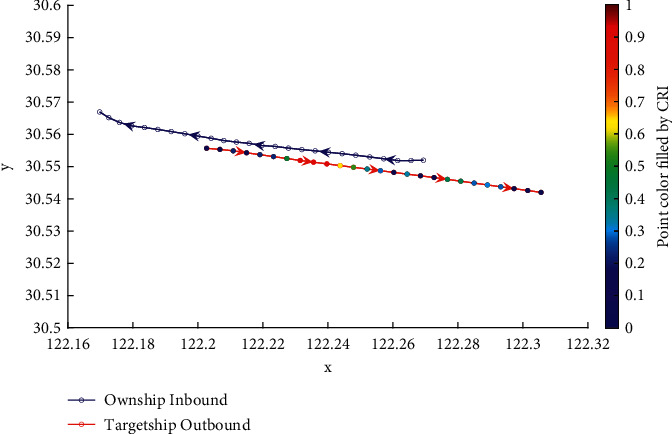
Visualization of collision risk between ship OS01 and ship TS05 with head-on situation.

**Figure 6:**
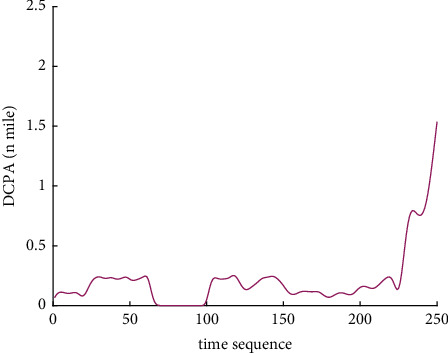
DCPA curve of single-ship encounter process.

**Figure 7:**
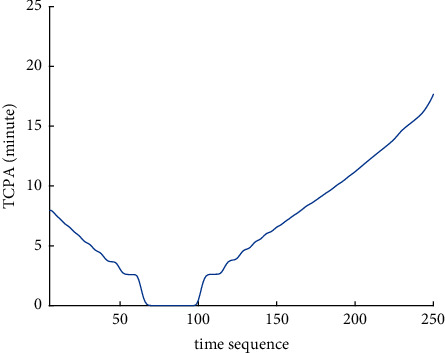
TCPA curve of single-ship encounter process.

**Figure 8:**
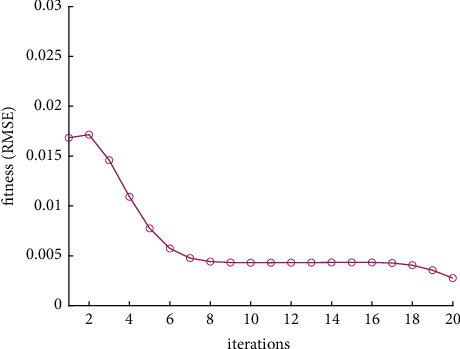
Fitness curve of PSO-LSTM training process.

**Figure 9:**
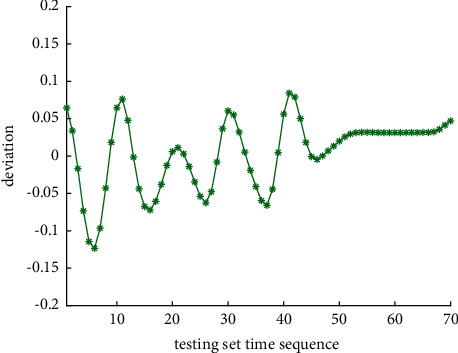
CRI deviation curve of PSO-LSTM prediction.

**Figure 10:**
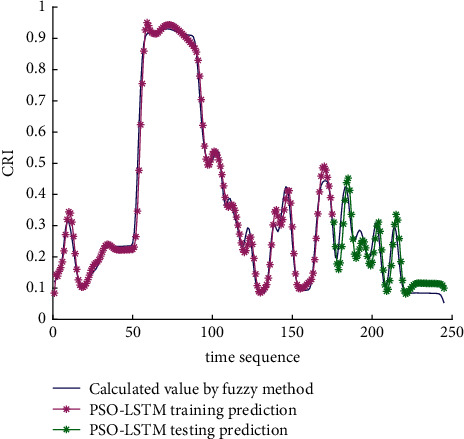
Predictive results of CRI in training and testing.

**Figure 11:**
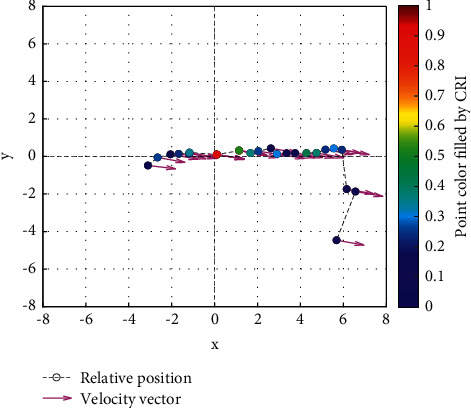
Visualization of collision risk prediction of ship TS05 in relative coordinates of own ship center.

AIS data are collected with an interval time of 6 seconds during the encounter between ship OS01 and ship TS05. In the experiment, approximately 255 groups of data is collected and processed, with the first 175 groups of data as training sets and the others as test sets. A complete group of data includes AIS data of the two ships, encounter parameters calculated by the formula, and CRI calculated by the fuzzy method. Taking 10 groups of data as time intervals, corresponding to one minute, are used in the rolling window. After the training and testing PSO-LSTM network, the experimental result curves of DCPA, TCPA and CRI and Fitness, etc., are smoothed and the relevant curves are drawn as follows:

Analysis of the single-ship experiment results is as follows:

1. Figure 5 reflects the track of ship OS01 and ship TS05 in the encounter process. The arrow represents the direction of the track, and the colour filled in track dots of the target ship TS05 reflects CRI which is obtained according to the Fuzzy calculation method. It can directly reflect the change in collision risk in the encounter process. The redder the colour is, the more dangerous it is.
2. From Figure 5, the risk index of the target ship leaving the port turns red many times during the encounter with its own ship, indicating that the collision risk of the target ship increases significantly during the close encounter. At this time, the ship's pilot needs to be vigilant and pay close attention to the dynamics of the target ship in the encounter until it passes clear.
3. According to Figures 6 and 7, in the process of ship encounter, DCPA and TCPA decrease and then increase rapidly as the two ships get closer and farther away. The corresponding collision risk also increases and then decreases.
4. The Fitness curve in the training process of PSO-LSTM network is reflected in Figure 8, which is also the RMSE convergence process. The experiment shows that the deviation is rapidly reduced and stable in the iterative optimization process of PSO, and PSO-LSTM network training process is effective.
5. The deviation of PSO-LSTM test prediction results is reflected in Figure 9, which indicates that the CRI prediction accuracy of PSO-LSTM is high, and the overall deviation range is ±0.1.
6. Figure 10 intuitively reflects the prediction effect of PSO-LSTM model by comparing the original data, the predicted data of training process and the predicted data of testing process. The experimental results show that PSO-LSTM model has strong prediction ability and good prediction effect. It also shows that PSO-LSTM model can be well adapted to ship collision prediction in single-ship encounter situation.
7. Figure 11 shows the visualization of CRI prediction results. The changes of CRI in ship encounter can be intuitively seen in relative coordinates, where the arrow is the velocity vector of the target ship TS05. From the figure, it can be easily found that when ship TS05 approaches ship OS01, its CRI increases rapidly, and the colour of the position point of ship TS05 turns red rapidly.
8. After the experiment, the pilots are consulted about their feelings. In the process of meeting with the target ship TS05, the pressure is obviously felt when approaching, which is due to the close distance and the large ship size. The psychological danger perception of the driver at that time is consistent with the situation reflected by the CRI curve in this test.

### 4.2. Multi-Ship Encounter Situation Experiment

**Figure 12:**
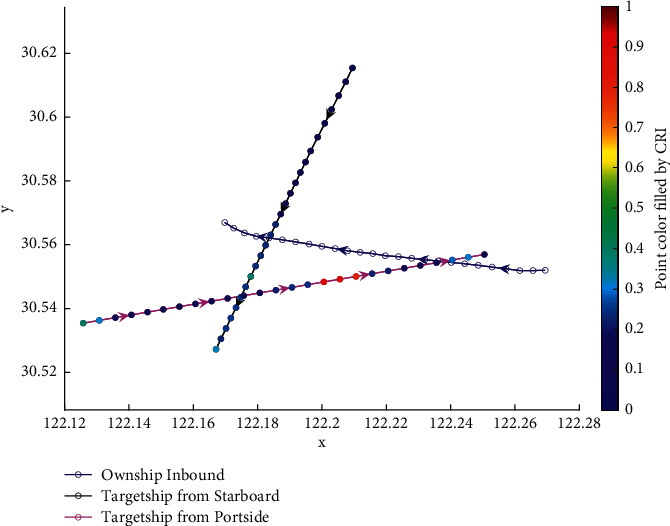
Visualization of collision risk in multi-ship encounter situation.

**Figure 13:**
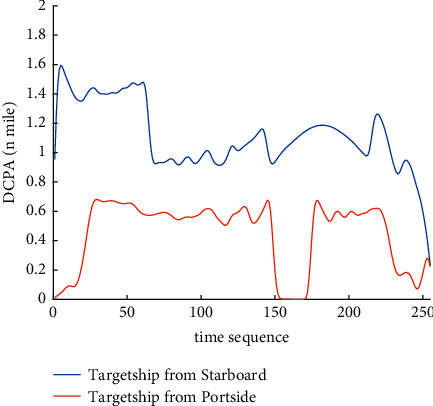
DCPA curve of multi-ship encounter process.

**Figure 14:**
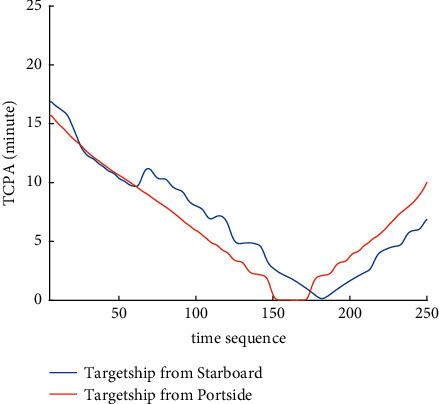
TCPA curve of multi-ship encounter process.

**Figure 15:**
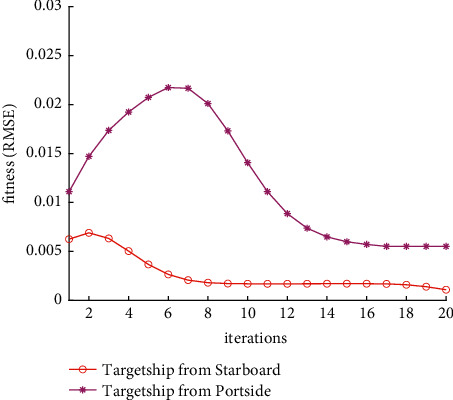
Fitness curve of PSO-LSTM training process.

**Figure 16:**
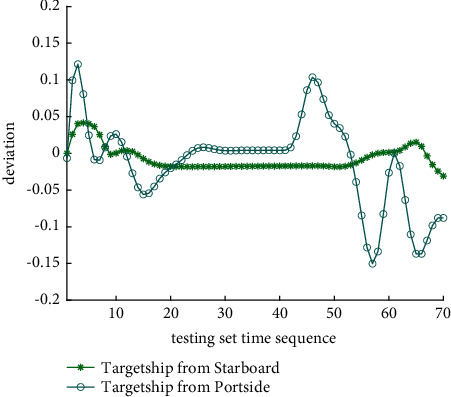
CRI deviation curve of PSO-LSTM prediction.

**Figure 17:**
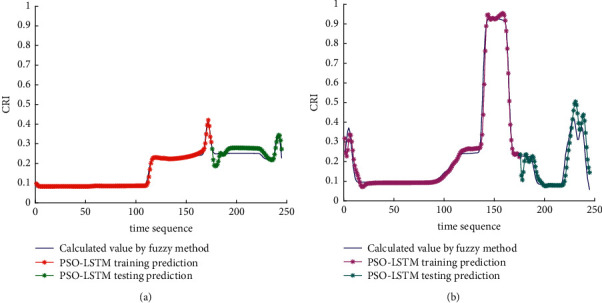
Predictive results of CRI in training and testing. (a) Target ship TS08 from Start broad. (b) Target ship TS19 from Portside.

**Figure 18:**
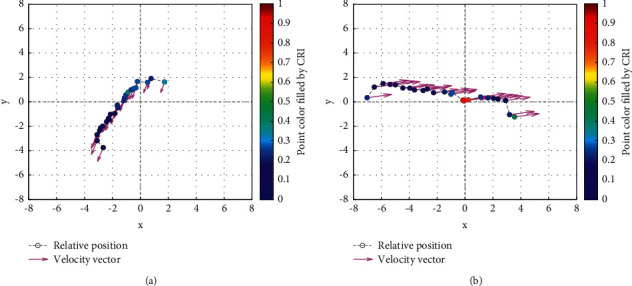
Visualization of collision risk prediction in relative coordinates of own ship center. (a) Target ship TS08 from Start broad (b) Target ship TS19 from Portside.

References for the single ship test, the same test parameters are set except for the target ship. AIS data are collected with an interval time of 6 second during the encounter between ship OS01 and ships TS08 and TS19. In the experiment, approximately 255 groups of data is collected and processed, with the first 175 groups of data as training sets and the others as test sets. Taking 10 groups of data as time intervals, corresponding to one minute, are used in the rolling window. After the training and testing PSO-LSTM network, the experimental result curves of DCPA, TCPA, CRI and Fitness, etc., are smoothed and the relevant curves are drawn as follows:

The analysis of the multi-ship experiment results is as follows:

1. Figure 12 reflects the track of the ship and the target ships TS08 and TS19 in the encounter process. According to the colour changes of the position points, the CRI of the target ship TS08 is relatively stable, and the risk increases slightly when the target ship crosses and approaches the own ship from the starboard side, indicating that the collision risk is small. By comparison, the CRI of the target ship TS19 changed relatively greatly, resulting in several sudden increases. In the process of the target ship crossing and approaching from the port side, the corresponding position points turned red several times, indicating the danger of collision. The pilot needs to pay attention to the target ship TS19 until it passes and clears. In the experiment, the overall collision risk of the target ship from the port side is greater than that of ship from the starboard.
2. Figures 13 and 14 reflect the change characteristics of the DCPA and TCPA, respectively, in the process of multi-ship encounter. By comparison, it is found that with the approach and departure of the target ship, the TCPA decreases roughly and then increases rapidly, while the DCPA shows the characteristics of fluctuation.
3. In Figure 15, the Fitness curves of target ships TS08 and TS19, also known as RMSE curves, can converge, indicating that the training process of PSO-LSTM is effective and that the deviations are gradually reduced under the action of PSO optimization. The Fitness curves show that starboard ship is faster than port ship in PSO optimization and that deviation convergence is faster.
4. In Figure 16, the CRI prediction curve analysis of the test set shows that the prediction accuracy of PSO-LSTM is high, with the starboard prediction deviation range of ±0.05 and port prediction deviation range of ±0.15.
5. Figures 17(a) and 17(b) show the performance of PSO-LSTM in the training and testing processes. There is a high degree of fit between the results and the original data, and the predicted results are close to the actual ones. The results show that PSO-LSTM can effectively and accurately predict ship collision risk, and adapt to the CRI prediction of multi-ship encounter situation.
6. In Figures 18(a) and 18(b), the CRI change process of target ships TS08 and TS19 is visualized.  Figure 18(a) shows that when ship TS08 passed the bow of ship OS01, CRI rapidly and gradually decreased and remained at a low level, indicating a low overall risk. From Figure 18(b), during the encounter between the target ship TS19 and the own ship, there is a very close process, and CRI turns red many times, so there is a collision risk, which should be considered.
7. After the experiment, the pilots are consulted about their feelings. During the course of the ship encounter, they clearly felt that the ship from the portside was more urgent, closer and more dangerous, which is consistent with CRI and its prediction.

## 5. Conclusion

Collision risk analysis is an important for ship navigation safety assurance. It is necessary to make collision risk forecast and forewarning. CRI, namely ship collision risk index, is mostly considered with DCPA, TCPA and other parameters in the process of ship encounter. These parameters are time sequences, which in performance before and after time are interrelated, and the corresponding CRI also shows time correlation characteristics. The rolling prediction mechanism and time memory ability of LSTM model can well reflect the related characteristics. PSO algorithm can optimize the hyperparameters of LTSM model, and better prediction results can be obtained. In this paper, through single-ship encounter and multi-ship encounter tests, PSO-LSTM model was used to obtain good results in CRI prediction. The Fitness curve of PSO in the training process converges rapidly, and the prediction deviation range of the test results is small. The results show that PSO-LSTM model can adapt to complex encounter situations and show high efficiency and accurate ship collision risk prediction.

In the future, we will consider using the multi-ship prediction capability of PSO-LSTM model to access real-time AIS data, and train a more powerful network through massive data learning to provide assistance for collision risk prediction for ship pilots earlier and in a timely manner. In addition, combined with the external environment, the deep learning model will be more effective in predicting and warning ship collision hazards. It is believed that with the development of navigation intelligence in the future, more intelligent deep learning methods will be more widely applied in the navigation field to ensure navigation safety and promote the development of the unmanned vessel and navigation intelligence.

## Figures and Tables

**Table 1:** Main parameters of the ships in experiments.

AIS number	Attribute	Length (m)	Width (m)	Draught (m)	Ship type	Encounter situation
OS01	own ship	274.7	40	10	5000TEU container ship	—
TS05	Target ship	347	42.8	14.4	8000TEU container ship	Head-on
TS08	Target ship	81.8	13.8	4.4	3000 ton river ship	Starboard crossing
TS19	Target ship	263.2	32.2	8.5	3000TEU container ship	Portside crossing

**Table 2:** Environmental parameters during the simulation test.

Wind direction (degree)	Wind scale	Flow direction (degree)	Flow velocity
45	Level 4	310	1 knot

## Data Availability

The datasets used in this paper are available from the corresponding author upon request.
